# Modeling of hepatitis B virus infection spread in primary human hepatocytes

**DOI:** 10.1128/jvi.00927-25

**Published:** 2025-08-04

**Authors:** Zhenzhen Shi, Masataka Tsuge, Nicholson Collier, Yasue Takeuchi, Takuro Uchida, Carolyn M. Rutter, Yuji Teraoka, Susan L. Uprichard, Yuji Ishida, Chise Tateno, Jonathan Ozik, Harel Dahari, Kazuaki Chayama

**Affiliations:** 1 The Program for Experimental & Theoretical Modeling, Division of Hepatology, Department of Medicine, Stritch School of Medicine, Loyola University Chicago 828441, Maywood, Illinois, USA; 2 Liver Center, Hiroshima University Hospital 68272, Hiroshima, Japan; 3 Department of Gastroenterology, Graduate School of Biomedical & Health Sciences, Hiroshima University 592189 , Hiroshima, Japan; 4 Consortium for Advanced Science and Engineering, University of Chicago 2456, Chicago, Illinois, USA; 5 Decision and Infrastructure Sciences, Argonne National Laboratory 1291, Lemont, Illinois, USA; 6 Hepatitis Information Center, The Research Center for Hepatitis and Immunology, National Center for Global Health and Medicine 417951 , Ichikawa, Chiba, Japan; 7 Division of Travel Medicine and Health, Research Center for GLOBAL and LOCAL Infectious Diseases, Oita University 12995, Yufu, Japan; 8 Department of Gastroenterology, Faculty of Medicine, Oita University 13235 , Yufu, Japan; 9 Fred Hutchinson Cancer Research Center, Hutchinson Institute for Cancer Outcomes Research, Biostatistics Program, Public Health Sciences Division 7286 , Seattle, Washington, USA; 10 Gastroenterology, National Hospital Organization Kure Medical Center and Chugoku Cancer Center 634487, Kure, Japan; 11 PhoenixBio Co., Ltd., Higashi-Hiroshima, Japan; 12 Hiroshima Institute of Life Sciences, Hiroshima, Japan; 13 RIKEN Center for Integrative Medical Sciences 198286, Yokohama, Japan; Wake Forest University School of Medicine 12279, Winston-Salem, North Carolina, USA

**Keywords:** viral hepatitis, hepatitis B virus, primary human hepatocytes, Myr-preS1 treatment, agent-based modeling

## Abstract

Chronic hepatitis B virus (HBV) infection poses a significant global health threat, causing severe liver diseases including cirrhosis and hepatocellular carcinoma. We characterized HBV DNA kinetics in primary human hepatocytes (PHHs) over 32 days post-inoculation (p.i.) and modified our *in-vivo* agent-based modeling (ABM) to gain insights into the HBV lifecycle and spread *in vitro*. Parallel PHH cultures were mock-treated or treated with HBV entry inhibitor Myr-preS1 (6.25 µg/mL) was initiated 24 h p.i. In untreated PHH, three viral DNA kinetic patterns were identified: (i) an initial decline, followed by (ii) rapid amplification and (iii) slower amplification/accumulation. In the presence of Myr-preS1, viral DNA and infected cell numbers in phase 3 were effectively blocked, with minimal to no increase. This suggests that phase 2 represents viral amplification in initially infected cells, while phase 3 corresponds to viral spread to naïve cells. The ABM reproduced well the HBV kinetic patterns observed and predicted that the viral eclipse phase lasts between 18 and 38 h. After the eclipse phase, the viral production rate increased over time, starting with a slow production cycle of 1 virion per day, which gradually accelerated to 1 virion per hour after 3 days. Approximately 4 days later, virion production reached a steady state production rate of 4 virions/h. The estimated median efficacy of Myr-preS1 in blocking HBV spread was 91% (range: 90–92%). The HBV kinetics and the predicted estimates of the HBV eclipse phase duration and HBV production cycles in PHH are similar to those predicted in uPA/SCID mice with human livers.

**IMPORTANCE:**

While primary human hepatocytes (PHHs) are the most physiologically relevant culture system for studying HBV infection *in vitro*, a comprehensive understanding of HBV infection kinetics and spread in PHH is lacking. In this study, we characterize HBV viral kinetics and modify our *in vivo* agent-based modeling (ABM) to provide quantitative insights into the HBV production cycle and viral spread in PHH. The ABM provides an estimate of the HBV eclipse phase duration, HBV production cycles, and Myr-preS1 efficacy in blocking HBV spread in PHH. The results resemble those predicted in uPA/SCID mice with human livers, demonstrating that estimated HBV infection kinetic parameters in PHH *in vitro* mirror those observed in the *in vivo* HBV infection chimeric mouse model.

## INTRODUCTION

An estimated 254 million people are chronically infected with the hepatitis B virus (HBV), with 1.2 million new infections reported annually (1). HBV infection leads to approximately 1.1 million deaths each year, primarily due to HBV-related liver disease such as cirrhosis and hepatocellular carcinoma (1). Current treatment options for chronic hepatitis B include antiviral therapy with nucleoside/nucleotide analogs (NUCs) and/or pegylated interferon alpha (2, 3). Although these treatments can suppress HBV replication and slow the progression of liver disease, they do not cure the infection. To develop more effective antiviral therapies aimed at eradicating HBV, it is essential to better understand the HBV lifecycle and spread (4).

Primary human hepatocytes (PHHs) are well known for their high susceptibility to HBV infection and their utility in molecular studies of the HBV lifecycle (5). However, only a few studies have utilized PHH cultures to examine HBV viral kinetics, and comprehensive data on viral dynamics and the kinetics of HBV spread remain scarce (6–8).

Mathematical modeling of HBV infection and treatment plays an important role in elucidating the interactions between HBV and its host (9–13). Recent modeling of HBV propagation from infection to steady state in humanized mice has emphasized the significance of viral production cycles within individual cells for understanding the complex, multiphasic kinetics of HBV infection *in vivo* (14). However, to date, there has been no modeling of HBV spread in PHH.

In this study, we aimed to characterize the kinetics of HBV infection by monitoring DNA at frequent time points and tracking HBV spread by monitoring the number of infected cells over 32 days post-inoculation (p.i.) in PHH in the absence or presence of the HBV entry inhibitor Myr-preS1. Modeling was performed by adapting our recently developed agent-based modeling (ABM) of HBV infection in humanized mice to simulate HBV-host interactions in PHHs (14), incorporate media replenishment and calibrate the model with measured HBV DNA and HBV-infected cell number. We identified three distinct phases of both extracellular and intracellular HBV DNA kinetics. The ABM predicted that Myr-preS1 treatment is highly effective (91%) in blocking viral spread, as evidenced by an extremely slow increase in the third phase of both extracellular and intracellular HBV DNA kinetics. The model estimates also demonstrated that HBV infection dynamics in PHH *in vitro* closely mirror that observed in the *in vivo* humanized liver chimeric mouse model.

## RESULTS

### HBV infection of PHH *in vitro* reveals three extracellular HBV DNA kinetic phases

**Fig 1:**
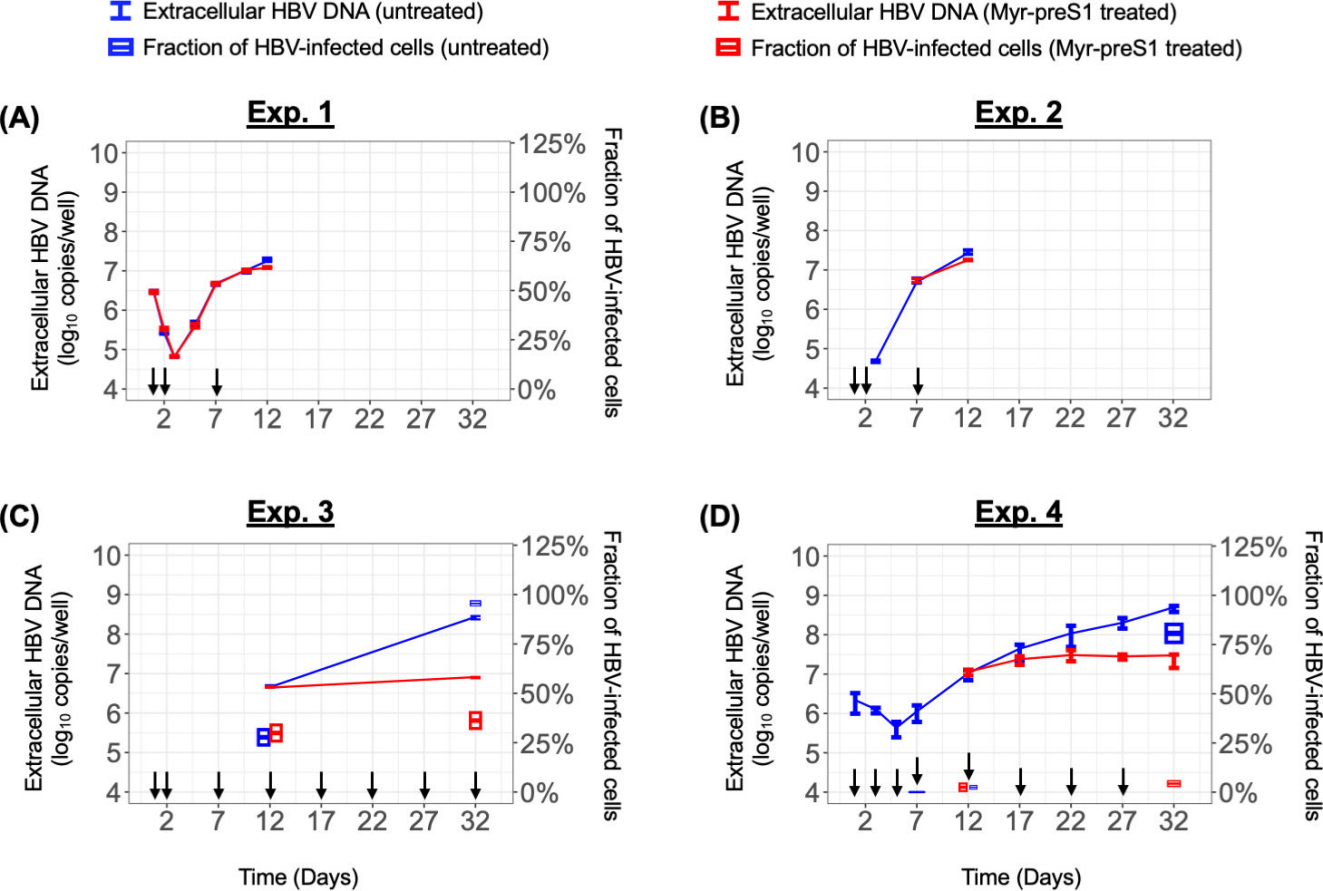
Measured extracellular HBV DNA and percentage of HBV-infected cells in Exps. 1–4 (**A–D**). A solid line represents the median value of extracellular HBV DNA in the control group (blue) and under Myr-preS1 treatment (red). Error bar denotes the minimal and maximal measurements of extracellular HBV DNA. Boxplot represents the percentage of HBV-infected cells (average ± SD). Arrows indicate the media changes/replenishment.

To characterize HBV kinetics in PHH up to 32 days p.i., four independent experiments were performed. Briefly, PHHs were seeded in collagen-coated 24-well or 48-well plates and were treated with 10 HBV genome equivalent per cell (GEq/cell) (Exps. 1–3) or 1 HBV GEq/cell (Exp. 4) for 1 day. One day after inoculation, PHHs were cultured in the medium with or without Myr-preS1. PHH and culture medium were collected at several time points. Extracellular HBV DNA was measured via qPCR, revealing three kinetic phases consisting of a rapid decline (phase 1), a rapid increase (phase 2), and a slow increase (phase 3) (Fig. 1, blue lines; Table S1). In Exp. 1, there was a rapid decline phase with a slope of −0.82 log_10_/day (*t*_1/2_ = 8.8 hr, 95% CI, [7.7, 10.2]) until day 3 p.i., followed by a rapid increase phase with a slope of ~0.46 log_10_/day (*t*_2_ = 15.6 h, 95% CI, [14.7, 16.7]) between days 3 and 7 p.i., followed by a slower increase phase with a slope of ~0.12 log_10_/day (*t*_2_ = 59.9 h, 95% CI, [53.3, 68.3]) between days 7 and 12 p.i. (Fig. 1A). Similar to Exp. 1, the extracellular HBV DNA in Exp. 2 increased rapidly with a slope of ~0.51 log_10_/day (*t*_2_ = 14.2 h, 95% CI, [13.4, 15.0]) from days 3 to 7 p.i., followed by a slower increase with a slope of ~0.15 log_10_/day (*t*_2_ = 49.6 h, 95% CI, [41.8, 61.0]) from days 7 to 12 p.i. (Fig. 1B). In Exp. 3, a slow increase was seen from days 12 to 32 p.i. with a slope of ~0.09 log_10_/day (*t*_2_ = 82.5 h, 95% CI, [78.6, 86.7]) (Fig. 1C). In Exp. 4, which was initiated at lower GEq/cell to provide a detailed longitudinal kinetic picture. The initial decline had a slope of −0.18 log_10_/day (*t*_1/2_ = 40.1 h, 95% CI, [35.0, 46.9]) until day 5 p.i. This was followed by an increase with a slope of ~0.17 log_10_/day (*t*_2_ = 42.8 h, 95% CI, [40.54, 45.29]) between days 5 and 17 p.i. Thereafter, extracellular HBV DNA had a slower increase with a slope of ~0.07 log_10_/day (*t*_2_ = 102.7 h, 95% CI, [93.3, 114.3]) from days 17 to 32 p.i. (Fig. 1D). Thus, as observed in our previous *in vivo* HBV infection in chimeric mice, the kinetics of extracellular HBV DNA was delayed in Exp. 4 (GEq/cell = 1) compared to the Exps. 1–3, where the inoculum was a log_10_ higher (GEq/cell = 10).

### Intracellular HBV DNA kinetics during infection of PHH *in vitro* mirror the same three phases as extracellular HBV DNA

**Fig 2:**
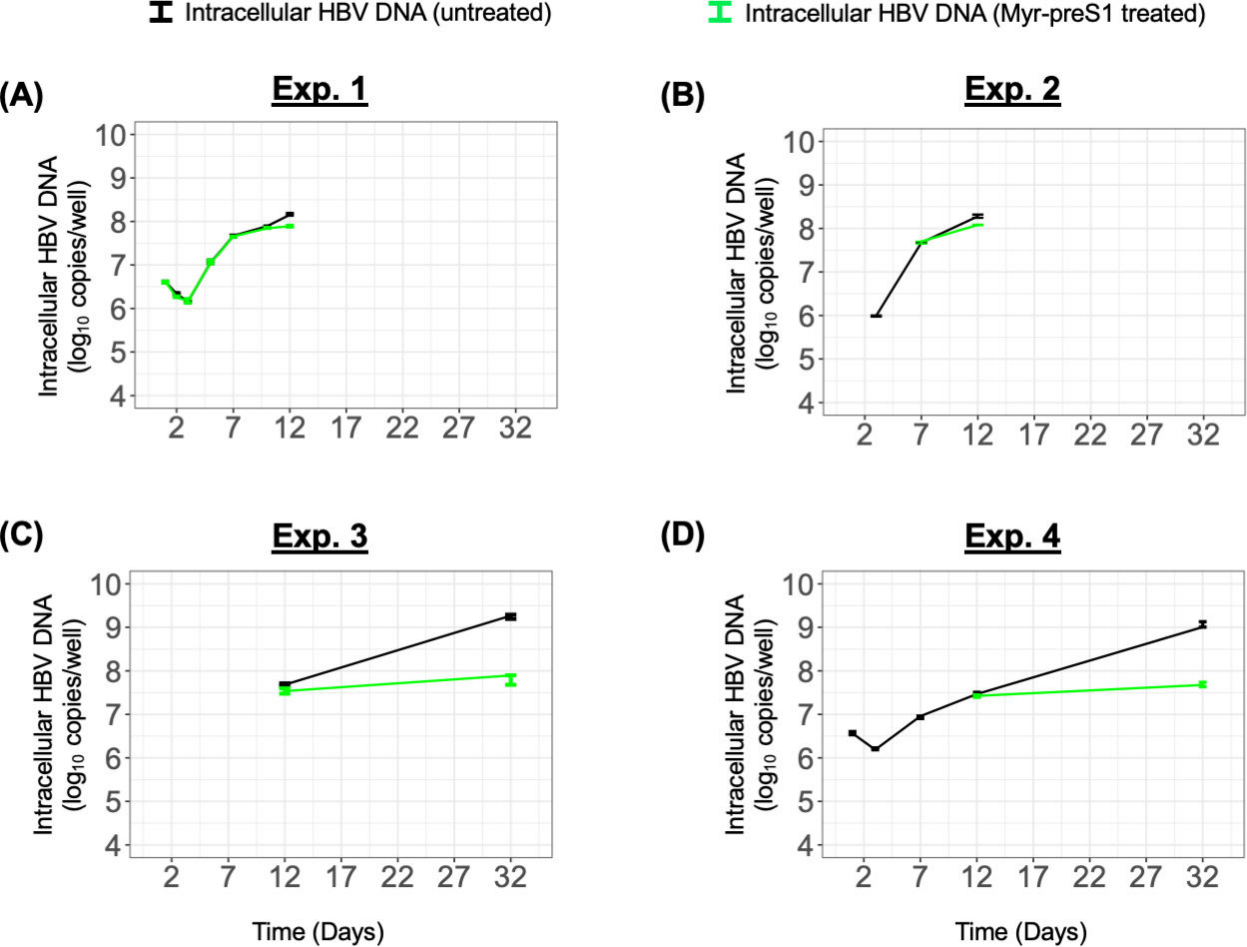
Measured intracellular HBV DNA in Exps. 1–4 (**A–D**). Solid line represents the median value of intracellular HBV DNA in the control group (black) and under Myr-preS1 treatment (green). Error bar denotes the minimal and maximal measurements of intracellular HBV DNA.

To characterize HBV intracellular kinetics in PHH up to 32 days p.i., cell lysates were collected at each time point in the four experiments and total HBV DNA was measured via qPCR. Analogous to what was observed for extracellular HBV DNA, three phases of cell-associated HBV DNA kinetics were identified: rapid decline (phase 1), rapid increase (phase 2), and slow increase (phase 3) (Fig. 2, black lines and Table S2). In Exp. 1, cell-associated HBV DNA declined with a slope of −0.23 log_10_/day (*t*_1/2_ = 31.7 h, 95% CI, [28.4, 35.8]) from days 1 to 3 p.i. Thereafter, intracellular HBV DNA increased rapidly with a slope of ~0.38 log_10_/day (*t*_2_ = 19.1 hr, 95% CI, [17.2, 21.5]) from days 3 to 7 p.i., followed by a slower increase phase with a slope of ~0.10 log_10_/day (*t*_2_ = 74.3 h, 95% CI, [63.6, 89.4]) from day 7 onwards (Fig. 2A). Likewise, in Exp. 2, the intracellular HBV DNA rapidly increased with a slope of ~0.42 log_10_/day (*t*_2_ = 17.2 h, 95% CI, [16.9, 17.4]) from days 3 to 7 p.i., followed by a slower increase with a slope of ~0.12 log_10_/day (*t*_2_ = 59.4 h, 95% CI, [54.0, 66.0]) until day 12 p.i. (Fig. 2B). Similar to the slower amplification phase of Exps. 1 and 2, the intracellular HBV DNA increased slowly with a slope of ~0.08 log_10_/day (*t*_2_ = 92.1 h, 95% CI, [86.8, 98.2]) from days 12 to 2 p.i. in Exp. 3 (Fig. 2C). Although initiated at a lower GEq/cell of 1, we observed a similar kinetics of cellular HBV DNA in Exp. 4: first phase decline slope of −0.18 log_10_/day (*t*_1/2_ = 39.4 h, 95% CI, [33.0, 49.1]) until day 3 p.i., followed by a fast increase with a slope of ~0.19 log_10_/day (*t*_2_ = 39.0 h, 95% CI, [35.8, 42.8]) between days 3 and 7 p.i., followed by a slower increase with a slope of ~0.08 log_10_/day (*t*_2_ = 87.3 h, 95% CI, [82.2, 93.0]) from days 7 to 32 p.i. (Fig. 2D).

### Myr-preS1 treatment affects the third phase of both intracellular and extracellular HBV DNA kinetics

To determine the effect of blocking NTCP-mediated HBV entry in the four experiments performed, parallel cultures were treated with Myr-preS1 to block NTCP-mediated HBV entry starting 24 h after HBV inoculation. Under Myr-preS1 treatment, extracellular HBV DNA kinetics in Exp. 1 followed the same three phases: (phase 1) rapid decline with a slope of −0.93 log_10_/day (*t*_1/2_ = 7.8 h, 95% CI, [7.2, 8.4]) until day 3 p.i.; (phase 2) fast increase with a slope of ~0.46 log_10_/day (*t*_2_ = 15.7 h, 95% CI, [14.4, 17.3]) between days 3 and 7 p.i.; and (phase 3) a slower increase with a slope of ~0.09 log_10_/day (*t*_2_ = 84.8 h, 95% CI, [67.0, 115.6]) from day 7 onwards (Fig. 1A, red line). However, the last phase viral increase appeared slower and/or plateaued under Myr-preS1 treatment (Fig. 1A, red line), reduced from the ~0.12 log_10_/day (*t*_2_ = 59.9 h, 95% CI, [53.3, 68.3]) increase observed in the absence of Myr-S1 (Fig. 1A, blue line). The same pattern was observed in Exp. 2, with the slope between days 7 and 12 p.i. reduced from ~0.15 log_10_/day (*t*_2_ = 49.6 h, 95% CI, [41.8, 61.0]) to 0.10 log_10_/day (*t*_2_ = 69.7 h, 95% CI, [59.8, 83.5]) in the presence of Myr-preS1 (Fig. 1B, blue vs red line). Looking at days 12–32 p.i. in Exp. 3, there was a much slower increase in extracellular HBV DNA in the presence of Myr-preS1 (slope = 0.01 log_10_/day; *t*_2_ = 583.6 h, 95% CI, [490.9, 719.5]) compared to untreated cultures (slope = 0.09 log_10_/day; *t*_2_ = 82.5 h, 95% CI, [78.6, 86.7]) (Fig. 1C). Likewise, in Exp. 4, the third phase amplification was remarkably slower in the presence of Myr-preS1 (slope = 0.02 log_10_/day; *t*_2_ = 426.4 h, 95% CI, [324.7, 621.1]) compared to that observed in the untreated cultures (slope = 0.07 log_10_/day; *t*_2_ = 102.7 h, 95% CI, [93.3, 114.3]) (Fig. 1D).

To determine how blocking NTCP-mediated HBV entry in the four experiments impacts intracellular HBV DNA kinetics, cell lysates were harvested from the Myr-preS1-treated cultures in all four experiments and cell-associated HBV DNA was measured via qPCR. Under Myr-preS1 treatment, intracellular HBV kinetics in Exp. 1 follow three phases: (i) rapidly decline with a slope of −0.34 log_10_/day (*t*_1/2_=20.95 h, 95% CI, [18.54, 24.08]) until day 3 p.i.; (ii) fast increase with a slope of ~0.37 log_10_/day (*t*_2_ = 19.5 h, 95% CI, [17.6, 22.0]) between days 3 and 7 p.i.; (iii) slow increase with a slope of ~0.05 log_10_/day (*t*_2_ = 147.8 h, 95% CI, [119.2, 194.6]) from day 7 onwards. While phases 1 and 2 were equivalent to the untreated cultures, phase 3 had a considerably lower slope under Myr-preS1 treatments. The same pattern was observed in the other experiments. In Exp. 2, intracellular HBV kinetics had a slower increase under Myr-preS1 treatment with a slope of 0.08 log_10_/day (*t*_2_ = 91.0 h, 95% CI, [79.4, 106.6]) between days 7 and 12 p.i. In Exp. 3, an extremely slow increase during phase 3 was observed under Myr-preS1 compared to untreated PHH (slope = ~0.01 log_10_/day; *t*_2_ = 501.20 h, 95% CI, [282.56, 2215.51] vs ~0.08 log_10_/day; *t*_2_ = 92.10 h, 95% CI, [86.76, 98.14]) (Fig. 1C). In Exp. 4, the phase 3 slope under Myr-preS1 was 0.01 log_10_/day; *t*_2_ = 551.9 hr, 95% CI, [407.8, 853.8] compared to the untreated culture phase 3 slope ~0.08 log_10_/day (*t*_2_ = 87.3 h, 95% CI, [82.2, 93.0]) (Fig. 2D and Table S2).

### Effect of Myr-preS1 reveals that the third phase of HBV infection in PHH *in vitro* represents viral spread

Because Myr-preS1 is expected to block HBV spread to new PHH, we fixed parallel cultures at the indicated time points in Exps. 3 and 4 to quantify the percentage of HBV-infected cells in untreated and Myr-preS1-treated cultures (Fig. 1, blue vs red boxplots, respectively). In Exp. 3 on day 12 p.i., the mean percentage of HBV-infected cells in Myr-preS1-treated and untreated cultures was equivalent (29.9 ± 4.1% vs 27.7 ± 3.9%, respectively) (Fig. 1C). However, the percentage of HBV-positive cells was statistically lower under Myr-preS1 treatment compared to the untreated group on day 32 p.i. (36.2 ± 4.1% vs 95.6 ± 0.4%, respectively) (Fig. 1C). Similarly, in Exp. 4, there was no difference in the percentage of HBV-infected cells between Myr-preS1-treated and untreated cells on day 12 p.i. (1.8 ± 1.9% vs 2.0 ± 1.0%, respectively), but there was a significant difference in the percentage of HBV-infected cells on day 32 p.i. (4.3 ± 1.4% vs 80.7 ± 4.6%) (Fig. 1D). Taken together, this demonstrates that phase 3 primarily represents NTCP-dependent HBV spread.

### Model reproduces the kinetics of HBV infection observed in PHH and suggests high Myr-preS1 efficacy in blocking HBV spread

**Fig 3:**
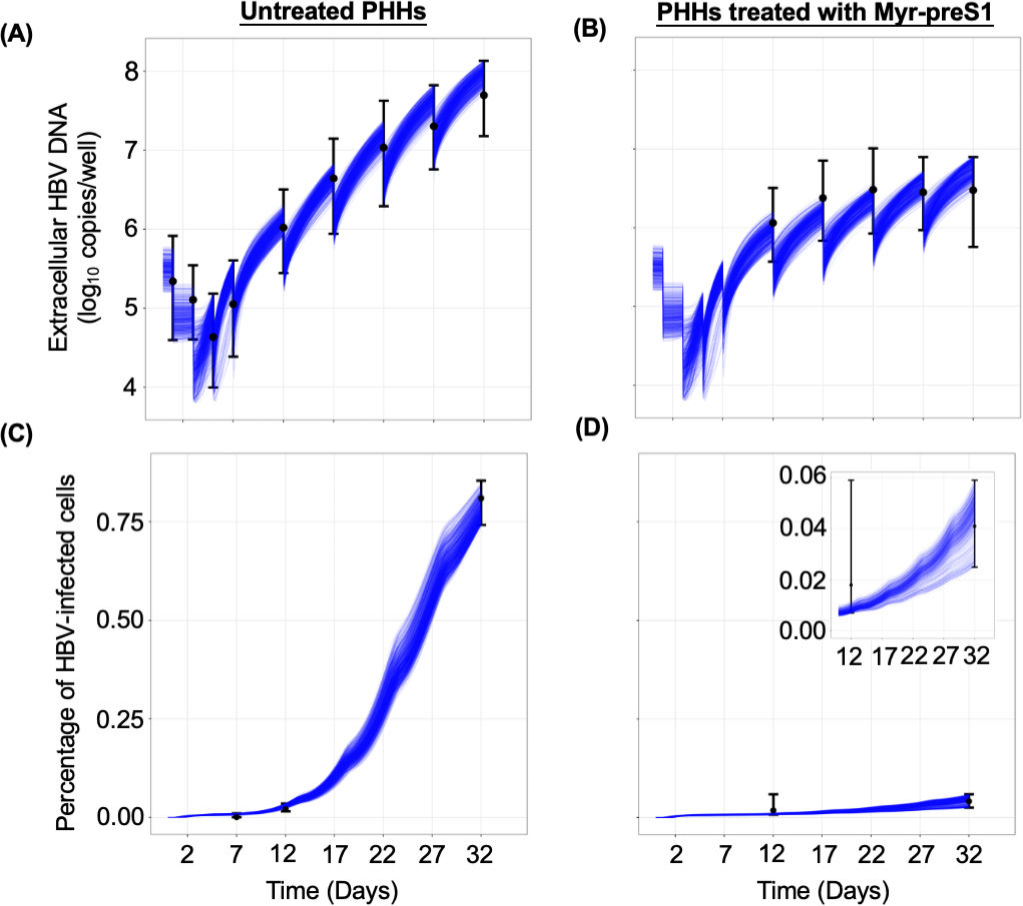
ABM calibration by fitting to Exp. 4. (**A–D**). ABM simultaneous calibrations (blue solid curves) with measured extracellular HBV DNA (top) and percentage of HBV-infected PHH (bottom) in Exp. 4. Left panels (A and C): Untreated. Right panels (B and D): Myr-preS1 treated. The ABM calibrations were done using the IMABC algorithm. Black dots are the median empirical calibration target values (i.e., extracellular HBV DNA) for each time point, error bars are the minimal and maximal bounds for the empirical targets, and blue lines represent 1,000 IMABC posterior outputs, all of which are contained within all of the empirical calibration bounds.

To capture the kinetics of HBV *in vitro* in PHH, we adapted our recently developed ABM and calibrated it with extracellular HBV data measured in Exp.4. Because Exp. 4 has the most complete HBV infection kinetics within a single experiment, we assessed whether the model could fit the HBV kinetics observed in Exp. 4, and the model reproduced well the extracellular HBV DNA multiphasic pattern (Fig. 3A and B) observed as well as the percent infected cell data (Fig. 3C and D). In addition, the oscillatory pattern of extracellular HBV DNA kinetics predicted in the ABM reflected well the media changes when the extracellular HBV DNA dramatically declined (Fig. 3A and B). The ABM was calibrated, resulting in sampled joint posteriors (Fig. S1, upper and lower rectangles) and marginal parameter distributions (Fig. S1, diagonal rectangles) of all the model parameters, both non-intervention and Myr-preS1 intervention parameters. We used a calibration stopping criterion of 1,000 effective sample size within the final calibration target bounds. These results reveal the parameter ranges for HBV infection and production and their high probability regions within those ranges that correspond to simulated target values consistent with the empirical targets. They also depict correlations between the infection-associated parameters (e.g., between ρ and β).

**Fig 4:**
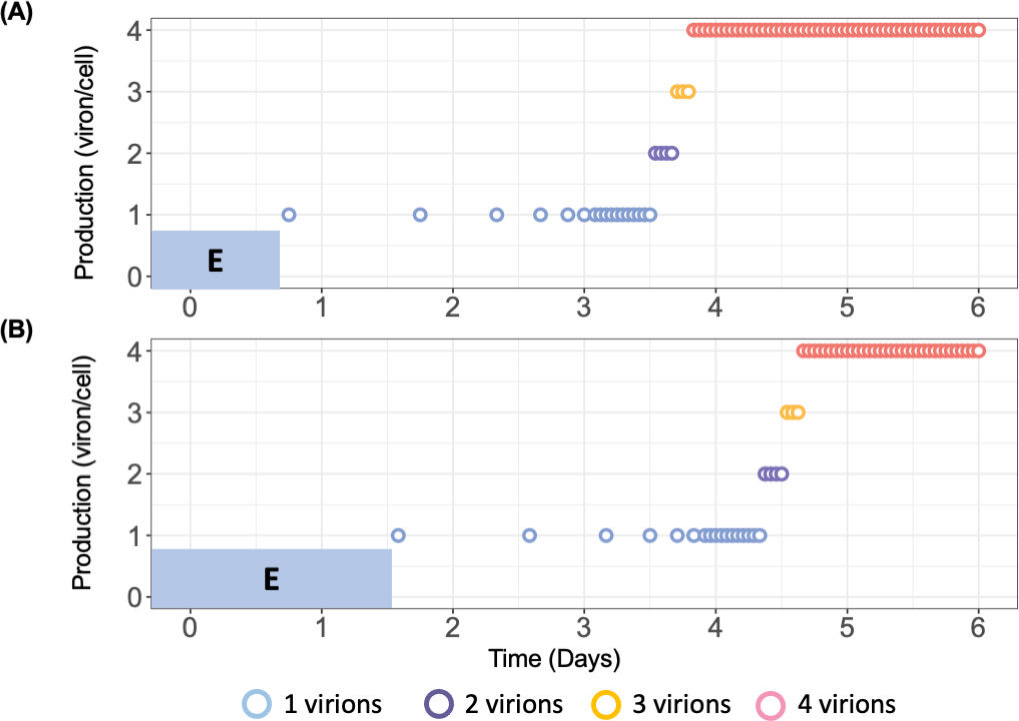
ABM prediction of HBV virion production cycles in PHHs. Blue-shaded “E” represents minimal eclipse phase (**A**) and maximal eclipse phase (**B**) for PHHs. Each PHH has a randomized eclipse phase followed by a consistent virion production pattern starting with 1 virion/day. As virus resources accumulate, the production cycle shortens. Production also increases to 2, and then three virions before reaching steady state of 4 virions/h. The magnitude of virion production was calculated using equation 1 and the time between each production cycle was calculated using equation 2. The parameter values (α, P_st_, γ, δ, and ω) used to calibrate equation 1 and equation 2 were the median level of estimated parameters (Table S4).

These modeling results estimate that the viral intracellular eclipse phase lasts between 12 and 45 h (Table 1). This eclipse phase ends and the P phase begins when the model predicts an increase in the intracellular viral production characterized by faster production cycles that release larger amounts of virus (Fig. 4). Specifically, during the first 2.5 days p.i., the model predicted that infected PHH have a long production cycle of 1 virion per day, but by 4 days p.i., infected PHH produce virions faster with a rate of 2 virions per hour. During the eclipse stage, virion production/release is minimal, so extracellular HBV DNA levels are observed to decrease consistent with the media removal/replenishment occurring at days 1 and 3 p.i. After day 4 p.i., the virion production in each cell reaches a steady-state production rate of 4 virions per hour leading to an increase in virion release and infection of new PHH that explains the fast extracellular HBV DNA increase observed between days 5 and 17 p.i. Then, as the number of target PHH decreases, the extracellular HBV DNA increase slows, consistent with the third phase of slower amplification. Again, looking at Exp. 4, the model recapitulated the much slower increase in extracellular HBV DNA from days 12 to 32 p.i. under Myr-preS1 treatment (Fig. 3B and D). Based on the calibration, the ABM predicts a median Myr-preS1 efficacy of 91.0% (first quantile [Q1]: 90% to third quantile [Q3]: 92%) to be achieved after a median 54 h (Q1: 40 h to Q3: 65 h) post-treatment initiation, suggesting a high efficacy of Myr-preS1 treatment in blocking HBV spread at an early stage of infection.

**TABLE 1:** IMABC calibration for fitting Exp. 4 with and without Myr-preS1

Category	Parameter description	Symbol [unit]	Prior simulations and preliminary fits (uniform)		IMABC calibration^*c*^	
			[Min–Max]	Source	Median	IQR [Q1–Q3]
Initial condition	Initial uninfected PHH number	*T*_0_ [cell]	[38,000–42,000]	Expert opinion^*a*^	39,900	[38,874–41,040]
	Initial viral load	*V*_0_ [virion]	[15,924–100,475]	Expert opinion^*a*^	30,662	[24,779–37,093]
Infection	Fraction of virion that are infectious	*ρ*	[0.145–0.58]	(14)	0.28	[0.19–0.33]
	Infection rate	*β* [cell/h]	[0.0001–0.0025]	(14)	0.0009	[0.0005–0.0011]
Eclipse phase	Min eclipse phase	*Ω*_min_ [h]	[5–36]	(14)	18	[12–26]
	Max eclipse phase	*Ω*_max_ [h]	[18–50]	(14)	38	[30–45]
Production (Eq. 1 and Eq.2)	Initial production cycle length	*δ* [h]	[15–30]	(14)	23.41	[19.62–27.19]
	Decay constant	*ω* [h^−1^]	[0.3–0.97]	(14)	0.54	[0.50–0.60]
	Number of cycles to reach 50% of *P*_st_	*α* [h]	[10–30]	(14)	21	[15–26]
	Steepness of the production curve	*γ* [h^−1^]	[0.1–0.9]	(14)	0.35	[0.26–0.48]
	Virus production at steady state	*P*_st_ [virion/cell/h]	[2–5]	(14)	4	[3–4]
Media change	Removal rate	Rr	[0.01–1]	Educational fits^*b*^	0.75	[0.68–0.77]
Myr-preS1 treatment	Efficacy of Myr-preS1	*η*	[0.5–1]	Educational fits^*b*^	0.91	[0.90–0.92]
	Time at Myr-preS1 taking effect	*t*_eff_ [h]	[24–72]	Educational fits^*b*^	54	[40–65]

^*a*^ Our experimentalists suggest that initial uninfected PHH number (*T*_0_) in an individual well is from 3.8 × 10^5^ to 4.2 × 10^5^. To improve the computational efficiency, we used 1/10 of the actual number which is the range of 3.8 × 10^4^ to 4.2 × 10^4^ in the ABM to model the virus spread in the culture media. The initial viral load (*V*_0_) is 4.0 × 10^5^ per well which was rescaled to 4.0 × 10^4^ to conform to the scaled-down PHH number. In addition, 0.4 log_10_ was added to the lower and upper bounds of each viral measurement to account for experimental variations so the final range for the initial viral load is from 15,924 (4.2 log_10_) to 100,475 (5 log_10_).

^*b*^ The range was determined based on preliminary model fittings using AnyLogic optimization tool.

^*c*^ Each parameter was sampled based on a uniform distribution (prior distribution) to implement IMABC. IQR represents the difference between the 25th and 75th percentiles of the marginal posterior distribution of parameters. All IMABC fits are shown in Fig. 3.

## DISCUSSION

In this study, we characterized the viral kinetics of HBV infection and assessed viral spread over a period of 32 days in PHH. We identified three distinct phases in both extracellular and intracellular HBV DNA kinetics: a first rapid decline, a second rapid increase, and a third slow increase phase. We recently reported (15) that extracellular HBV DNA is stable in media; therefore, the first rapid decline phase likely represents HBV entry into PHH and virus removal during early media replenishment. Our model calibration results (Fig. 3C) indicate that only a small fraction (0.3%) of PHHs were infected during the first 2 days after HBV inoculation, compared to an estimated 75% (IQR: 68–77%) of virions being removed from the media (Table 1). This supports the idea that the initial rapid extracellular HBV decline phase is primarily caused by replenishment of media as recently assumed in our previous report (15). Modeling results (Fig. 4A and B) predict that virion production per infected PHH peaks at approximately 4 virions/cell/h around day 5 p.i., which aligns with the dramatic rise in extracellular HBV DNA starting at that time (Fig. 1D). We speculated that the slower phase III HBV increase results as the target cells become more limiting. Notably, the second rapid and third slow increase phases observed in PHH closely resemble the two amplification phases (phases 4 and 6) seen in the humanized mouse model (14). We show that the ABM reproduces well the measured viral DNA kinetic and spread data by assuming a cyclic nature of viral production, characterized by an initial slow but increasing rate of HBV production in individual cells, consistent with the modeling findings during acute HBV infection in humanized mice (14). The ABM also predicts that Myr-preS1 treatment was highly effective (91 ± 1%) in blocking viral spread 30-h post-treatment initiation (IQR: 16 and 41 h), as evidenced by an extremely slow increase in the third phase of both extracellular and intracellular HBV DNA kinetics.

Although extracellular and intracellular HBV DNA exhibited similar kinetic patterns across four experiments, a more detailed analysis of viral kinetics under varying HBV inocula dose revealed differences. First, the extracellular HBV DNA level in Exp. 1 declined approximately five times faster (−0.82 log_10_/day) than in Exp. 4 (−0.18 log_10_/day) (Table S1). Given that HBV degradation is relatively stable in media (15), the faster decline in Exp. 1 suggests a higher rate of HBV removal from the media during media replenishment (see Supporting Text A and Fig. S2B), which could result if a higher percentage of 10-fold less virus in the Exp.4 inoculum had more binding sites available to attach to the cells and the surrounding tissue culture plastic and thus avoid removal/collection. Second, the rapid increase phase of both extracellular and intracellular DNA in Exp. 1 was faster. Extracellular HBV DNA increased ~3 times faster (0.46 log_10_/day) compared to Exp. 4 (0.17 log_10_/day) (Table S1), and intracellular HBV DNA increased twofold faster than Exp. 1 (0.38 log_10_/day) compared to Exp. 4 (0.19 log_10_/day) (Table S2). Because viral amplification is exponential, the 10-fold higher inoculum HBV levels in Exp. 1 (GEq/cell = 10) compared to Exp. 4 (GEq/cell = 1) would be expected to result in higher viral uptake and lead to more rapid initial amplification of the virus and faster viral spread (see Supporting Text A and Fig. S2D).

To the best of our knowledge, HBV spread in PHH has not been investigated in previous studies. The lack of increase in extracellular HBV DNA, intracellular HBV DNA, and the number of infected cells during phase 3 in the presence of Myr-preS1 suggests that phase 2 reflects viral amplification within initially infected cells, while phase 3 represents the spread to naïve cells. Consequently, phase 3 of intracellular HBV amplification, in the absence of Myr-preS1, parallels the spread observed in extracellular HBV kinetics. The continued, though slower, increase in intracellular HBV levels (with a slope of 0.01–0.08 log_10_/day; Table S2) during phase 3 in the presence of Myr-preS1 suggests either incomplete inhibition of viral spread or that intracellular HBV levels had not yet reached steady state.

To better understand the HBV kinetics observed in PHH, we adapted our recently developed ABM by incorporating media replenishment and accounting for the impact of Myr-preS1 interventions on HBV spread. The estimated initial uninfected PHH of 39,900 (IQR: 38,874–41,040) cells (Table 1) was found close to the assumed 40,000 cells (Table S3). The estimated HBV inoculation of 30,662 (IQR: 24,779–37,093) virions suggests GEq/cell of ~0.8, which is somewhat smaller than the assumed GEq/cell of 1 (Table S3). We estimated several key parameters that reflect both the duration of the HBV eclipse phase and its subsequent production cycles. Interestingly, our results showed that the duration of the intracellular HBV eclipse phase in PHH ranges from 18 to 38 h, a more narrow and consistent range compared to the 7–50 h predicted for the chimeric mouse model (14), and in agreement with the ~22-h duration from HBV inoculation to the start of cccDNA accumulation estimated in PHH (15). The model’s estimate for virion production at steady state (i.e., *P*_st_) is identical in both systems, with approximately 4 virions per infected cell per hour in PHH and in mice. Other key parameters related to the viral production cycle, such as initial production cycle length (20–27 h in PHH vs 23–30 h in humanized mice), the number of viral production cycles to reach 50% of *P*_st_ (15–26 in PHH vs 14–30 in humanized mice), and the steepness of the production curves (0.50–0.60 h^−1^ in PHH vs 0.40–0.60 h^−1^ in humanized mice), also show similarities, further suggesting that HBV infection kinetics in PHH closely resemble those in the chimeric mouse model. This similarity surprisingly suggests that viral replication is similarly efficient in human hepatocytes *in vitro* and *in vivo* in the chimeric mice. While it is acknowledged that *in vitro* experiments lack many of the physiological interactions of *in vivo* systems—such as systemic signals from other organs and the immune system—*in vitro* models offer a valuable platform for analyzing the detailed dynamics of HBV replication, free from the confounding effects of such factors.

HBV infects hepatocytes by first attaching to heparan sulfate proteoglycans and subsequently binding to the HBV receptor, human sodium taurocholate co-transporting polypeptide (hNTCP). We employed treatment with Myr-preS1 entry inhibitor to block viral binding to hNTCP by competitive inhibition. To assess its efficacy in PHH, Myr-preS1 was administered starting 1 day post-inoculation to prevent the spread of HBV infection. The model predicts that in the presence of Myr-preS1, the fraction of HBV-infected cells increased very slowly, from approximately 2–7%, between days 17 and 32 p.i., suggesting that Myr-preS1 effectively interfered with HBV spread (Fig. 3D). The estimated median efficacy of Myr-preS1 treatment was 91%, which is highly effective, but not complete. The incomplete inhibition observed suggests that (i) higher dosages may be required to fully block HBV infection, (ii) HBV may interact with other receptors during cell entry (16), and/or (iii) additional spread mechanisms may exist (e.g., direct spread to adjacent cells, forming infected cell clusters [17]). The simplest explanation would be that Myr-preS1 peptide, like many inhibitors, is not 100% effective, but all these possible explanations are worth investigating experimentally to determine if complete inhibition of HBV spread can be achieved.

While the Myr-preS1 peptide (tradename, Bulevirtide [BLV]) is already approved in Europe, additional NTCP inhibitors are in development (18–20) and perhaps could be combined to achieve 100% inhibition of HBV uptake if all HBV spread is mediated by NTCP. Targeting of HBV entry factors that work with NTCP could work in the same manner (16, 21, 22). Alternatively, other types of spread inhibitors (i.e., targeting other cellular receptors or spread mechanisms) or even other aspects of the viral lifecycle may be required. Regardless of how it is achieved, preventing HBV spread is a critical goal in the global elimination of HBV. In particular, combining entry inhibitors like BLV with other antivirals would be expected to help prevent the infection of the new liver after transplant (23) and mother-to-child transmission at birth (24). Having shown here that HBV infection and spread in PHH is comparable to that observed in the uPA/SCID chimeric mouse suggests that optimal combination treatments could be assessed *in vitro* with mathematical models facilitating the analysis.

The lack of decline in intracellular HBV DNA under Myr-preS1 treatment suggests that HBV infection remains stable in the absence of re-infection, which also implies that cccDNA maintenance was not dependent on re-infection within this 32-day infection period. However, with the cccDNA half-life predicted to be approximately 40 days *in vitro* (25), longer-term experiments are needed to confirm these findings.

Our study has several limitations. First, we mainly used Exp. 4 for modeling since longitudinal data were available from time of infection until day 32 p.i. Since Experiments 1–3 had less frequent data sampling and different experiment durations, we combined Exps. 1 and 3 into one longitudinal data set and made exploratory modeling efforts to explain the differences in HBV kinetics at varying HBV input doses (Supplementary Text A and Fig. S2). Future experimental work with more frequent sampling points until 32 days p.i. is needed to compare HBV viral kinetics across different doses. Second, we did not model intracellular HBV DNA and/or cccDNA kinetics because the current model was adopted from the *in vivo* model (14), which was not designed to account for their dynamics. To gain a deeper understanding of intracellular HBV dynamics, an integration of the current ABM with our recent mathematical model that accounts for intracellular viral DNA and cccDNA kinetics (15) is warranted. Including intracellular HBV dynamics in future theoretical models would help to test and refine our understanding of HBV life cycle in general, and particularly the notion of HBV production cycles predicted by our current ABM model. Moreover, such integrated models could also help to investigate the mode of action of antivirals that were designed to target intracellular HBV life cycle (e.g., NUCs) and other investigational drugs such as nucleic acid polymers, short-interfering RNA, and capsid assembly modulators (26–28). Finally, PHH death was not incorporated into the ABM. Previous characterization of our PHH culture system demonstrated that although cell density slightly decreases by day 2 p.i., the number of hepatocytes then remains stable for at least 33 days (29). Similarly, in the present study, stable cell counts were observed from day 7 until day 32 p.i. (Fig. S3b) during which most of HBV spread occurred (Fig. 3C). Based on this observation and prior work assessing hepatic function in human hepatocytes isolated from humanized mice, we assumed that excluding PHH loss or death would not significantly affect the results. However, for longer-term experiments and/or monitoring HBV infection in PHH with evident loss of cell availability (e.g., due to HBV genotype), the inclusion of cell death and PHH loss may be necessary for accurately calibrating HBV kinetics in the model.

In conclusion, this study demonstrates that HBV DNA levels follow multiphasic replication kinetics in PHH and uses theoretical modeling to recapitulate observed kinetics to provide insights into HBV spread dynamics. Modeling the entry inhibitor Myr-preS1 inhibition measured data helped to mechanistically define the kinetic phases of infection and indicated that treatment effectively blocks HBV spread, although it does not prevent it completely. By comparing model predictions from both the *in vivo* humanized mouse model and the *in vitro* PHH system, we show that HBV infection dynamics in PHH closely mirror those observed in the uPA/SCID chimeric mouse model, suggesting that there are virtually no other factors in this mouse model that impact HBV spread. Future studies, including mathematical modeling of intracellular replication and cccDNA transcription, will provide deeper insights into HBV spread and HBV-host dynamics at the molecular level.

## MATERIALS AND METHODS

### PHH preparation

Commercially available cryopreserved human hepatocytes (BD Bioscience, lot 195, Hispanic, female, 2 years) were transplanted into cDNA-uPA/SCID mice via injection into the spleen. PHHs were isolated from the chimeric mice with humanized livers at 9–15 weeks after transplantation by a standard collagenase perfusion. To expand the cells, the isolated PHHs were serially transplanted into additional cDNA-uPA/SCID mice as previously described (30). Nine to fifteen weeks after the serial transplantation, PHHs were collected for *in vitro* culture and infection. PHHs were seeded in collagen-coated 24-well plates (Corning, Japan) at 2.0 × 10^5^ cells/well (Exp. 1) and 4.0 × 10^5^ cells/well (Exps. 2 and 4) or in 48-well plates at 1.6 × 10^5^ cells/well (Exp. 3) and incubated at 37°C with 5% CO_2_ in dHCGM medium (Table S3). The base of this medium is Dulbecco’s modified Eagle’ medium (Gibco, ThermoFisher Scientific) supplemented with 44 mM NaHCO_3_ (Wako Chemicals), 100 IU/mL Penicillin G (Invitrogen, ThermoFisher Scientific), 100 µg/mL Streptomycin (Invitrogen, ThermoFisher Scientific), 20 mM HEPES (Gibco, ThermoFisher Scientific), 10% FBS (Biosera), 15 µg/ml l-proline (Wako Chemicals), 0.25 µg/mL insulin (Sigma-Aldrich), 50 nM Dexamethasone (Sigma-Aldrich), 5 ng/mL Epidermal growth factor (Sigma-Aldrich), 0.1 mM l-ascorbic acid 2-phosphate (Wako Chemicals), and 2% Dimethyl sulfoxide (Sigma-Aldrich).

### HBV inoculum

HBV genotype C2 obtained from a chronic hepatitis B patient and named Hiroshima GtC CL3 (NCBI accession no. MH818373) was used as inoculum in this study. The virus was amplified in cDNA-uPA/SCID mice as previously described (30).

### HBV infection

**Fig 5:**
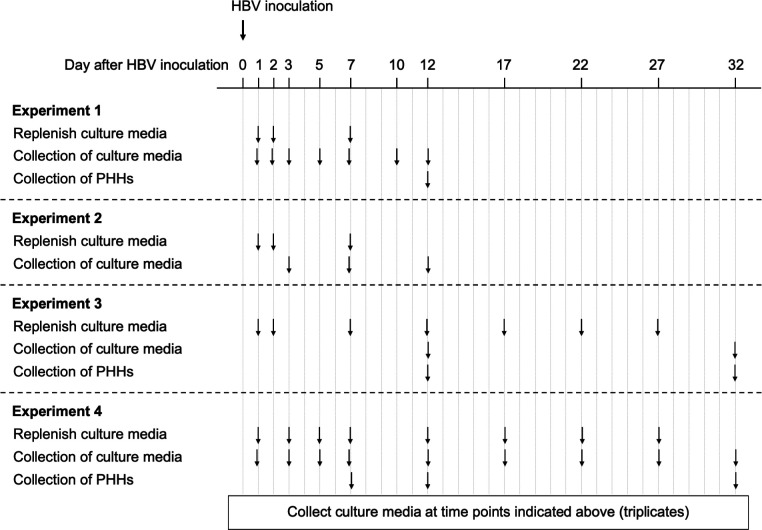
Experimental design for PHHs. PHHs were treated with an inoculum of 10 HBV GEq/cell (Exps. 1–3) or 1 HBV GEq/cell (Exp. 4) for 1 day, starting on day 0. Myr-preS1 (6.25 µg/mL) treatment was initiated 1 day after inoculation and then continued throughout the experiment. Extracellular HBV DNA and intracellular HBV DNA were measured in collected culture media at the indicated time points for each experiment. PHHs were harvested for estimating HBV-infected cells. During the infection, culture media were renewed after HBV inoculation or until the end of the experiment as indicated by arrows.

PHHs were treated with an inoculum of 10 HBV GEq/cell (Exps. 1–3) or 1 HBV GEq/cell (Exp. 4) for 1 day with 4% polyethylene glycol 8000 (PEG 8000). The inoculated cells were cultured with 200 or 500 µL of culture medium for 48-well and 24-well plates, respectively. Myr-preS1 (6.25 µg/mL) treatment was initiated 1 day after inoculation and then continued throughout the experiment. Extracellular HBV DNA and intracellular HBV DNA were measured in replicate wells at the indicated time points for each experiment. During the infection, culture media were renewed after HBV inoculation or until the end of the experiment as indicated by arrows in Fig. 5.

### Quantification of extracellular HBV DNA in culture medium

HBV DNA levels in culture media were quantified by qPCR as previously described (31). DNA was extracted using SMI TEST (Genome Science Laboratories, Tokyo, Japan) and dissolved in 20 µL of H_2_O. Real-time PCR analysis was performed using ABI Prism 7300 Sequence Detection System. Amplification was performed in a 25 µL reaction mixture containing SYBR Green PCR Master Mix (Applied Biosystems, Foster City, CA, USA), 200 nM of forward primer, 200 nM of reverse primer, and 1 µL of DNA or cDNA solution. After incubation for 2 min at 50°C, the sample was heated for 10 min at 95°C for denaturing, followed by a PCR cycling program consisting of 40 two-step cycles of 15 ss at 95°C and 60 s at 60°C. The lower detection limit of this assay was 2.3 log_10_ copies/mL. The primers used for extraction are as follows: forward 5′-TTTGGGGCATGGACATTGAC-3′ and reverse 5′-GAGTGCTGTATGGTGAGGTG-3′.

### HBV DNA isolation and analysis

DNA was extracted from the harvested PHH using SMITEST (Genome Science Laboratories) in accordance with the manufacturer’s instructions. Intracellular HBV DNA levels were quantified by qPCR and TaqMan PCR, respectively, using an ABI Prism 7300 (Applied Biosystems, Carlsbad, CA, USA), as previously described (32). Briefly, the concentration of purified DNA was measured by BioPhotometer 6131 (Eppendorf, Tokyo, Japan). Intracellular HBV DNA was quantified by qPCR using the same protocol and primers for extracellular HBV DNA quantification. For total HBV DNA, the forward primer (nucleotides [nt] 1,521–1,545: 5-GGGGCGCACCTCTCTTTACGCGGTC-3), reverse primer (nt 1,862–1,886: 5-CAAGGCACAGCTTGGAGGCTTGAAC-3), and TaqMan MGB probe (nt 1,685–1,704: 5-FAM-AACGACCGACCTTGAGGCAT-MGB-3) were utilized. PCR was performed using 100 ng of extracted DNA, TaqMan Fast Advanced Master Mix (Applied Biosystems), 300 nmol of each primer and 250 nmol of the probe. Amplification was performed as follows: 50°C for 2 min, then 95°C for 10 min, followed by 45 cycles of 95°C for 10 s, 58°C for 5 s, 63°C for 10 s, and 20 s at 72°C. The lower limit of detection was 100 copies/100 ng DNA.

### Estimation of HBV-infected cells

Immunostaining for HBsAg was conducted as previously described (30). At indicated times after inoculation, PHHs were fixed with 10% formalin neutral buffer solution for 10 min at room temperature. After washing two times with PBS, the fixed PHHs were treated with anti-HBsAg goat polyclonal antibody (bs-1557G; Bioss Inc, Woburn, MA, USA) as the first antibody and then treated with Alexa Fluor 488 donkey anti-goat IgG antibody (A11055; Thermo Fisher Scientific, MA, USA) as the second antibody. Subsequently, nuclei in PHH stained with Hoechst (Thermo Fisher Scientific). To analyze the percentage of HBsAg-positive PHH, five pictures were taken with a BZ-X700 microscope (Keyence, Osaka, Japan) and the number of PHH and HBsAg-positive PHH were counted (Fig. S3).

### Modeling HBV dynamics

**Fig 6:**
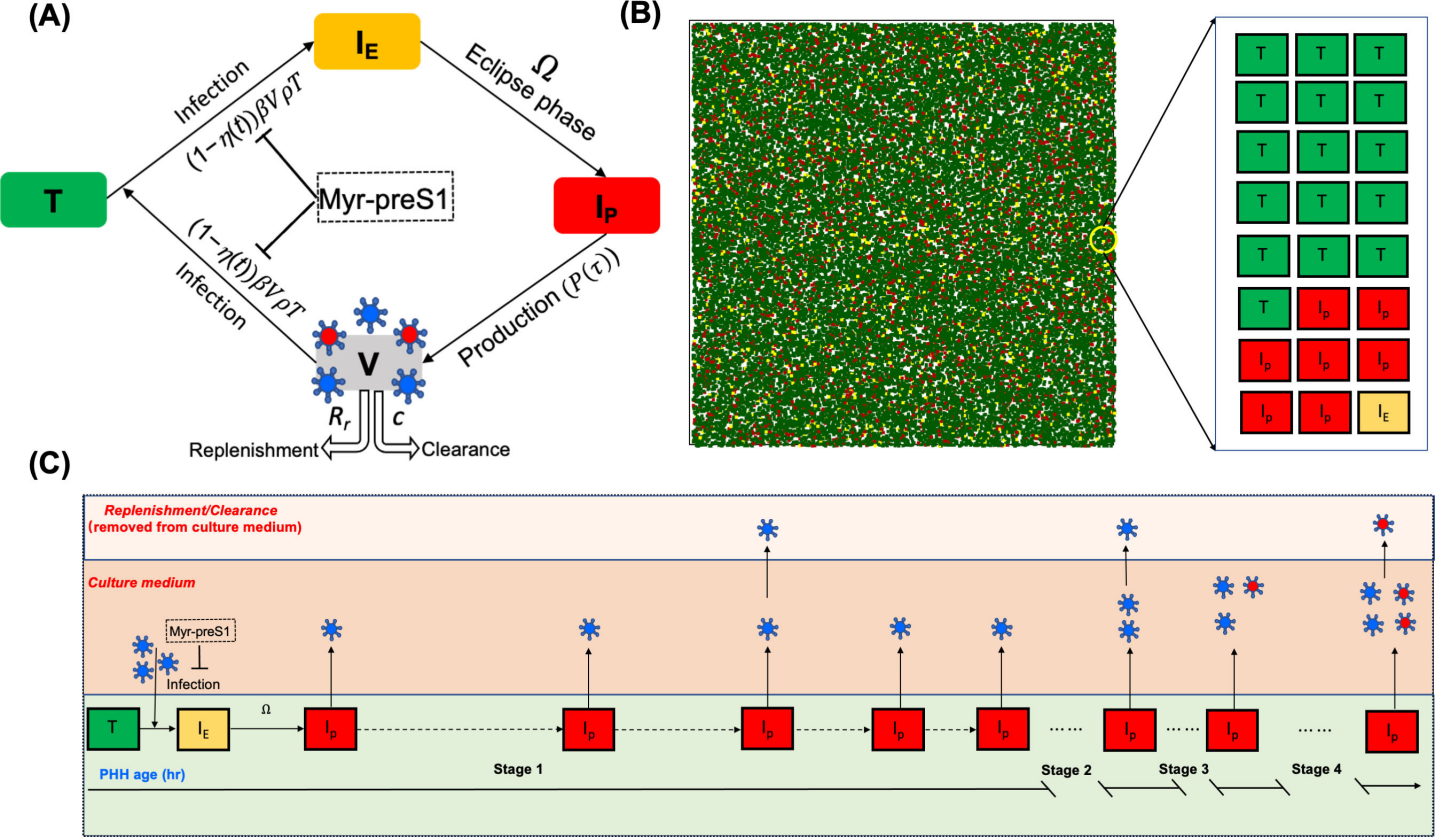
Conceptualization of the agent-based model. (**A**) The human hepatocytes can be only in one of the following three phases at a given time; *T* = uninfected cells which are termed as target or susceptible cells, *I*_E_ = HBV-infected cells in eclipse phase (i.e., not yet releasing virions), *I*_P_ = HBV-infected cells actively producing/releasing virions. Once *I*_E_ become *I*_p_, they produce free virus that can further infect *T*. The free virus in blood, *V*, is composed of infectious (red center virus) and non-infections virions (blue center virus). The parameter ρ represents the fraction of virions that are infectious, *β* represents the infection rate constant, *Ω* represents eclipse phase duration, *P*(τ) represents virion secretion from *I*_P_ (Eq. 1), *c* represents viral clearance from blood, *R_r_* represents the portion of virions removed during medium change/replenishment, and we assume no death/loss for PHHs in the culture media based on Fig. S3. The effectiveness of Myr-preS1 is *η* when the drug takes effect at *t*_eff_. (**B**) Simulated hepatocytes including *T* (green), *I*_E_ (yellow), and *I*_P_ (red) in ABM at 15 days after HBV inoculation. (**C**) Schematic diagram of a representative hepatocyte progressing through ABM. Each individual hepatocyte has its own infection kinetics followed by a randomized eclipse phase and viral production cycle as shown in Fig. 4.


To study HBV spread in PHH, we modified our recently developed ABM to describe HBV infection in humanized mice (14). Analogous to the published model, this ABM consists of two types of agents to account for the PHH and the extracellular virus. Here, the PHHs are represented by a two-dimensional grid of stationary cells while the virus is represented by freely diffusing agents intended to represent the amount of HBV in the culture medium. Each individual PHH can be in one of the following three discrete states: uninfected/susceptible PHH targets (*T*), infected PHH in an eclipse phase (*I*_E_), or productively infected PHH secreting progeny virus (*I*_P_) (Fig. 6). The ABM execution is again an iterative process where each iteration represents a discrete time step, each step = 1 h. For each iteration, uninfected/susceptible PHHs are infected and initially enter an eclipse phase (*I*_E_). After a random period of time following a uniform distribution of *U*(Ω_min_, Ω_max_), the infected PHH proceeded from the eclipse phase to the productive phase, and then they started to release newly generated progeny virus (i.e., *I*_p_). The magnitude and frequency of secreted virus are calculated using the following two equations to mimic the cyclic nature of the viral lifecycle within each cell (Eq.1 and Eq.2).

(Eq.1) $$P\left(\tau \right)=\frac{P_{st}}{1+e^{-(\tau -\text{α})}}$$

where P(τ) is number of virions produced by infected cells a  τ, $P_{st}$ is steady state virus production, α is number of cycles to reach to 50% of $P_{st}$, is steepness of the production curve, and τ is the production cycle.


(Eq.2) $$l_{\tau }=\delta e^{-\omega \tau }$$

where $l_{\tau }$ is interval between production cycle, τ is the production cycle, δ is scaling factor indicating the initial production cycle length, and ω is decay constant.

To account for the removal of virus-containing media and replenishment with fresh media, a removal/replenishment rate (*Rr*) was newly incorporated into the *in vitro* ABM which estimates the percentage of extracellular virus removed at each media change. To estimate the effect of the drug intervention on HBV spread, the following step function (Eq. 3) was also incorporated into the ABM


(Eq.3) $$\eta \left(t\right)=\left\{ \begin{array}{l} 0\;\;\;\;\;\;\;t<t_{eff}\\ \eta \;\;\;\;\;\;\;t\geq t_{eff} \end{array}\right.$$

where *η* is efficacy of Myr-preS1 and *t*_eff_ is time when Myr-preS1 starts to take effect. A schematic picture of the modified ABM is shown in Fig. 6.

#### Parameter estimation

We previously showed that the degradation of extracellular HBV DNA *in vitro* is extremely slow since the HBV is quite stable in the culture medium (33). Thus, in the ABM, we assumed a fixed virus clearance rate of 0.0004 h^−1^ (*t*_1/2_ = 72.2 days) in between the media changes. To estimate the remaining parameters, we applied the following two steps including preliminary AnyLogic fits and Incremental Mixture Approximate Bayesian Computation (IMABC) (34). Removal/replenishment parameter *Rr* was estimated to be in the range of (0.01–1) by fitting the ABM to the untreated PHH experimental data using AnyLogic optimization tool. Parameter ranges for efficacy of Myr-preS1 (*η*) and time when Myr-preS1 takes effect (*t*_eff_) were estimated by fitting the ABM to the experimental data under Myr-preS1 intervention. Parameter ranges for other parameters were determined based on the *in vivo* mouse model (14). By assuming uniform prior distributions for each parameter using the estimated ranges in the first step, we applied IMABC to obtain the posterior distributions of parameters.

#### Model calibration using IMABC

The ABM was calibrated by identifying parameters that result in model predictions that fit the experimental data. We utilized the IMABC algorithm (34) for calibration, implemented with the R IMABC package (35) and the EMEWS framework (36) on Argonne’s LCRC Bebop high-performance computing cluster. The IMABC algorithm works by adaptively constraining model output target bounds. The algorithm converges when a sufficient number of model parameters (effective sample size, i.e., 1,000 samples) are found to simulate targets within the final empirical target bounds. During model calibration, two sets of ABM targets were sequentially evaluated, corresponding to non-intervention and intervention data. The calibration algorithm first used the ABM to simulate non-intervention targets (i.e., untreated PHH). If the non-intervention ABM did not result in points within the non-intervention targets, the parameter set was rejected. For each parameter vector that produced simulated targets (Table S4) within the specified IMABC target bounds, the algorithm next evaluated fit to intervention targets from the Myr-preS1 intervention. Through this approach, the non-intervention and intervention ABM parameters were simultaneously estimated.

## Data Availability

Data are available on request from the authors.

## SUPPLEMENTAL MATERIAL

Supplemental material jvi.00927-25-s0001.docx Tables S1 to S4, Fig. S1 to S3, and Text A.

ASM does not own the copyrights to Supplemental Material that may be linked to, or accessed through, an article. The authors have granted ASM a non-exclusive, world-wide license to publish the Supplemental Material files. Please contact the corresponding author directly for reuse.
