# Supplementary material for: Modeling of hepatitis B virus infection spread in primary human hepatocytes
Source: J Virol. 2025 Aug 4;99(9):e00927-25. doi: 10.1128/jvi.00927-25 (PMC12455953; doi:10.1128/jvi.00927-25)
Supplement: Supplemental material — Tables S1 to S4, Fig. S1 to S3, and Text A. [file jvi.00927-25-s0001.docx]

# Supplemental Material

**Modeling of hepatitis B virus infection spread in primary human hepatocytes**

Zhenzhen Shi, Masataka Tsuge, Nicholson Collier, Yasue Takeuchi, Takuro Uchida, Carolyn M. Rutter, Yuji Teraoka, Susan L. Uprichard, Yuji Ishida, Chise Tateno, Jonathan Ozik, Harel Dahari, Kazuaki Chayama

**Table S1.** Detailed characterization of extracellular HBV kinetics under Exps. 1-4

| Exp.# | Phase 1  Slope (log_10_/d)  [95%CI] | | Phase 2  Slope (log_10_/d)  [95%CI] | | Phase 3  Slope (log_10_/d)  [95%CI] | |
| --- | --- | --- | --- | --- | --- | --- |
|  | Untreated | Myr-preS1 | Untreated | Myr-preS1 | Untreated | Myr-preS1 |
| Exp.1 | -0.82  [-0.94, -0.71] | -0.93  [-1.00, -0.86] | 0.46  [0.43, 0.49] | 0.46  [0.42,0.50] | 0.12  [0.11, 0.14] | 0.09  [0.06, 0.11] |
| Exp.2 | NA | NA | 0.51  [0.48, 0.54] | NA | 0.15  [0.12, 0.17] | 0.10  [0.09, 0.12] |
| Exp.3 | NA | NA | NA | NA | 0.09  [0.08, 0.09] | 0.01  [0.01, 0.02] |
| Exp.4 | -0.18  [-0.21, -0.15] | NA | 0.17  [0.16, 0.18] | NA | 0.07  [0.06, 0.08] | 0.02  [0.01, 0.02] |
| NA, not available; CI, confidence interval. | | | | | | |

**Table S2.** Detailed characterization of intracellular HBV kinetics under Exps. 1-4

| Exp. # | Phase 1  Slope (log_10_/d)  [95%CI] | | Phase 2  Slope (log_10_/d)  [95%CI] | | Phase 3  Slope (log_10_/d)  [95%CI] | |
| --- | --- | --- | --- | --- | --- | --- |
|  | Untreated | Myr-preS1 | Untreated | Myr-preS1 | Untreated | Myr-preS1 |
| Exp.1 | -0.23  [-0.25, -0.20] | -0.34  [-0.39, -30] | 0.38  [0.34, 0.42] | 0.37  [0.33, 0.41] | 0.10  [0.08, 0.11] | 0.05  [0.04, 0.06] |
| Exp.2 | NA | NA | 0.42  [0.41, 0.43] | NA | 0.12  [0.11, 0.13] | 0.08  [0.07, 0.09] |
| Exp.3 | NA | NA | NA | NA | 0.08  [0.07, 0.08] | 0.01  [0.00, 0.03] |
| Exp.4 | -0.18  [-0.22, -0.15] | NA | 0.19  [0.17, 0.20] | NA | 0.08  [0.08, 0.09] | 0.01  [0.01, 0.02] |
| NA, not available; CI, confidence interval. | | | | | | |

**Table S3** Initial conditions for each experiment

|  | Exp.1 | Exp. 2 | Exp.3 | Exp.4 |
| --- | --- | --- | --- | --- |
| Number of Hepatocyte (cells/well) | 2.0 x 10^5^ | 4.0 x 10^5^ | 1.6 x 10^5^ | 4.0 x 10^5^ |
| Plate  (well) | 24 | 24 | 48 | 24 |
| Volume of culture medium (uL/well) | 500 | 500 | 200 | 500 |
| GE/cell  (genome equivalents/cell) | 10 | 10 | 10 | 1 |

**Table S4.** Experimental data (simulated targets) for Exp. 4

| Day | Extra HBV-median [min, median, max] | Percentage of HBV infected cells [min, median, max] | |
| --- | --- | --- | --- |
| 1 | [4.595, 5.339, 5.915] | |  |
| 3 | [4.603, 5.106, 5.542] | |  |
| 5 | [3.994, 4.634, 5.184] | |  |
| 7 | [4.384, 5.050, 5.604] | | [0, 0.0005, 0.01] |
| 12 | [5.443, 6.021, 6.504] | | [0.015, 0.02, 0.035] |
| 17 | [5.941, 6.644, 7.146] | |  |
| 22 | [6.291, 7.034, 7.627] | |  |
| 27 | [6.758, 7.304, 7.823] | |  |
| 32 | [7.178, 7.696, 8.131] | | [0.742, 0.810, 0.854] |


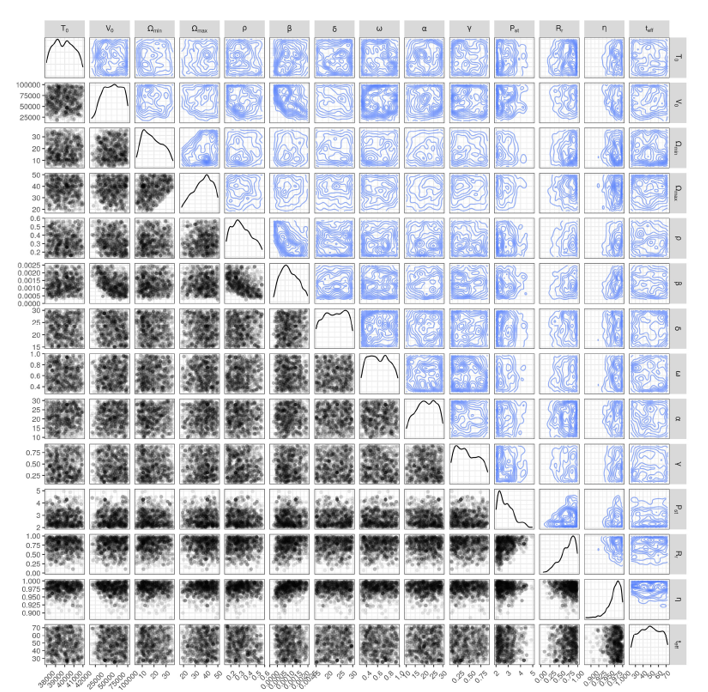


**Figure S1. Joint posterior distributions of non-intervention and Myr-preS1 intervention parameters from IMABC (1000 samples).** The upper (contour plots) and lower (scatter plots) triangles show the two-dimensional joint posterior distributions. The diagonal shows the marginal distributions of each parameter in **Table 1**.

**Supporting Text A:** **Exploratory mathematical modeling of combined measured data of Exp. 1 and Exp. 3.**

For model fitting purposes we have combined extracellular HBV measurements from Exp. 1 with those from Exp. 3. To align these measurements, we adjusted the extracellular HBV data from days 12 and 32 in Exp. 3 to the experimental conditions of Exp. 1. This adjustment was based on two factors: the size of the seeded culture well area and the volume of culture medium. The seeded area and culture medium volume in Exp.1 were 5.27 times larger than in Exp. 3, so all HBV measurements from Exp. 3 (on days 12 and 32) were multiplied by a factor of 5.27. After combining the data (**Fig. S2**, black error bars), the extracellular HBV kinetics show consistency with the three main HBV kinetic phases observed in Exp. 4: fast decline, fast increase, and slow increase. The percentage of HBV-infected cells ranged from 21.5% to 31.9% on day 12 and from 95.1% to 96.2% on day 32. We then used the best ABM parameter estimates from Exp. 4 (**Table 1,** main text) to fit the combined dataset, adjusting only the initial conditions (i.e., initial hepatocyte number and viral load, Table S3) and the media replenishment schedule (Fig. 5, revised main text) to match the combined experimental design (**Fig. S2A**).

To replicate the faster viral decline phase observed in Exp. 4 (~0.82 log/day vs. ~0.18 log/day in the combined dataset), a higher removal rate (parameter Rr) was required, which is not unexpected as this parameter is impacted by differences in the media change schedule between experiments and volume being exchanged (**Fig. S2B**). Additionally, one of the following predicted mechanisms needed to be incorporated into the ABM to reproduce well not only the extracellular HBV kinetics but also the percentage of HBV-infected cells of the combined dataset: (1) an increase in the fraction of infectious virus (**Fig. S2C**), (2) an increase in the infection spread rate (**Fig. S2D**), or (3) a decrease in the eclipse phase (**Fig. S2E**). Because the same virus was used in all experiments, predicted mechanism 1 would not be expected to be different between experiments. However, because virus amplification is exponential, a higher MOI would be expected to initially generate more virus and result in faster viral spread (predicted mechanism 2). We may reject predicted mechanism 3 because we previously demonstrated in the chimeric mice, that the eclipse phase was not affected after the dose of infection (ref (14) , main text).


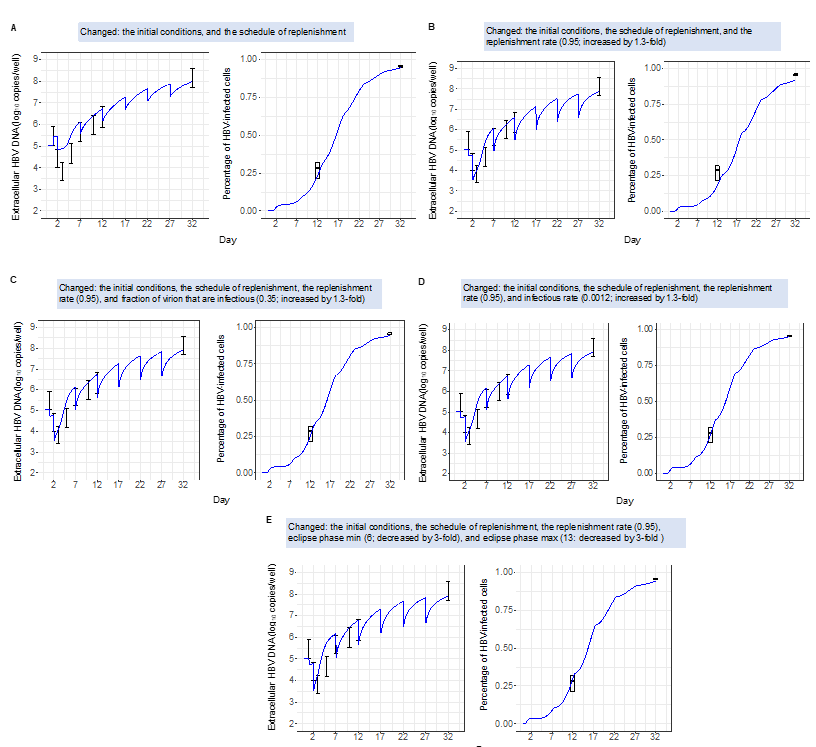
**Figure S2.** **ABM calibration for extracellular HBV DNA in the combined data set (Exp. 1 + Exp.3).** Black error bar and black boxplot represent extracellular HBV DNA measurements and the percentage of HBV-infected cells, respectively. Blue lines represent the ABM calibration. **(A)** ABM was calibrated by only changing the initial conditions and the schedule of replenishment (Fig. 5 and **Table S3**). Initial uninfected PHH number and initial viral load were set to 19000 and 109648, respectively. Culture media were renewed at 1, 2, 7, 12, 17, 22, and 27 days after HBV inoculation. **(B)** Besides the initial conditions and the schedule of replenishment, ABM was calibrated by changing the removal rate (parameter Rr). ABM can reproduce well the extracellular HBV DNA and percentage of infected cells by changing one of the following parameters (C): fraction of virion that are infectious; **(D)**: infection rate; and **(E)**: eclipse phase.


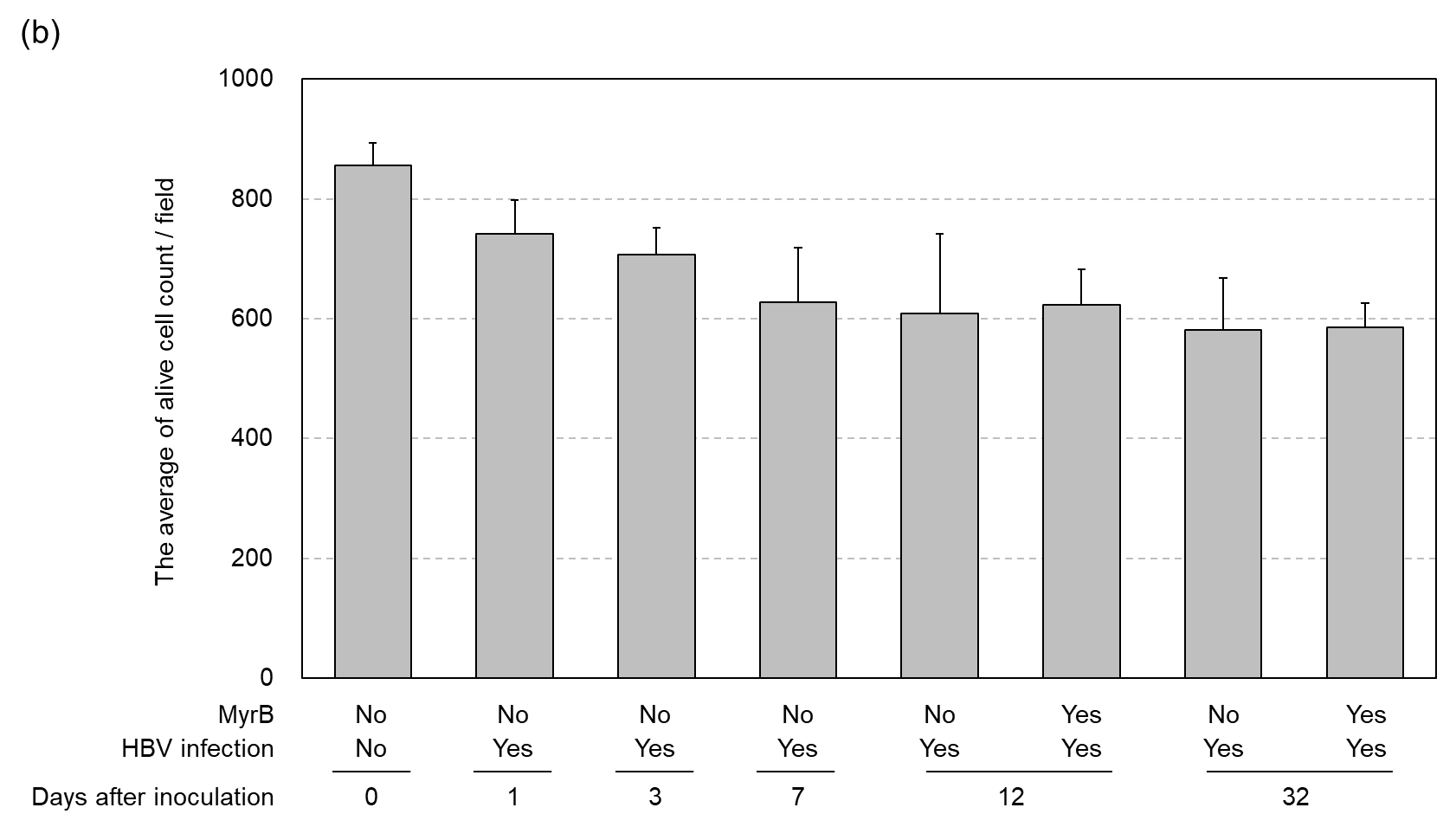

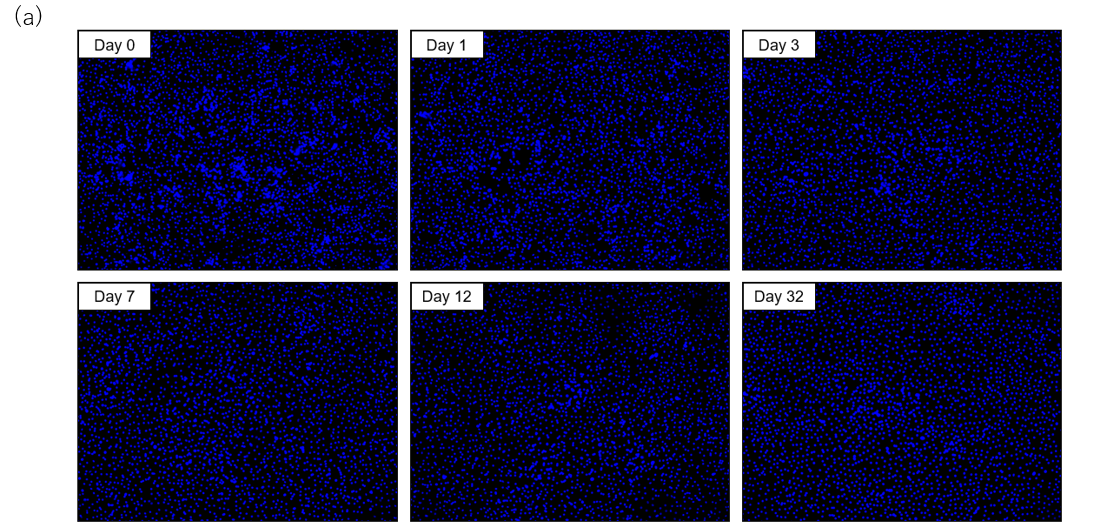


**Figure S3. PHH numbers present in cultures over time.** The cells were stained with Hoechst at each time point **(a)** and the stained cells were counted by BZ-X700 microscope **(b)**.
